# Tuberculosis manifesting with significant peripheral eosinophilia: A case report and review of literature

**DOI:** 10.1002/ccr3.8085

**Published:** 2023-10-23

**Authors:** Abhiram Rao Damera, Prakash Gupta, Shaheer Farooqi, Vivek Sanker, Arpita Mariam Mathews, Shreya Pampati, Manoj Reddy Allala, Tirth Dave

**Affiliations:** ^1^ Mediciti Institute of Medical Sciences Ghanpur Telangana India; ^2^ Team Erevnites India; ^3^ Virgen Milagrosa University Foundation College of Medicine San Carlos Philippines; ^4^ Department of Medicine Ziauddin University Karachi Pakistan; ^5^ Noorul Islam Institute of Medical Sciences Thiruvananthapuram Kerala India; ^6^ Mount Zion Medical College Pathanamthitta Kerala India; ^7^ Kamineni Institute of Medical Sciences Narketpally Telangana India; ^8^ Bukovinian State Medical University Chernivtsi Ukraine

**Keywords:** Mycobacterium, peripheral eosinophilia, pulmonary tuberculosis

## Abstract

**Key Clinical Message:**

Peripheral eosinophilia is a rare but potential sign of TB infection. Physicians should assess patients for TB, especially if they display related symptoms or risk factors, and consider TB as a differential diagnosis, especially in idiopathic cases.

**Abstract:**

Millions of new tuberculosis (TB) cases are reported annually. Peripheral eosinophilia is rare in tuberculosis. We describe a rare case of tuberculosis with a high peripheral eosinophil count. A 9‐year‐old male presented with fever, cough, and respiratory discomfort for a month. The patient's cough did not respond to treatment, along with weight loss and a loss of appetite. A physical examination revealed cervical lymphadenopathy and bilateral lung crepitations. A hematological investigation showed a high eosinophil count of 25,920 cells per cubic millimeter and medical imaging abnormalities consistent with TB. Some malignancies, allergies, and parasitic infections produce peripheral eosinophilia. However, medical literature rarely discusses TB‐induced eosinophilia. Several studies attribute it to mycobacterium antigen hyperreactivity. Eosinophilic release of cytotoxic chemicals may cause tissue damage, and TB patients' eosinophil levels may fluctuate. This case report emphasizes the need to investigate TB in peripheral eosinophilia patients after ruling out other explanations. Our patient benefited from early detection and anti‐TB medication. More studies are required to investigate the causes of TB eosinophilia and its consequences. A detailed medical history and physical examination are essential to diagnose and treat atypical presentations of TB.

## Graphical abstract

Chest x‐ray showing diffuse nodular calcifications in both lungs, innvolving all zones.

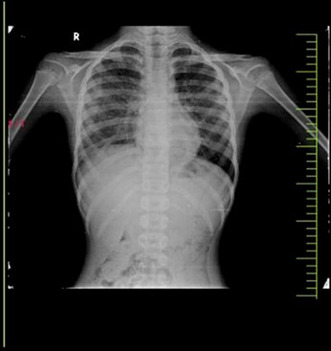

## 1 INTRODUCTION

Tuberculosis (TB) is still one of the most prevalent infections, especially in the developing world. The World Health Organization (WHO) estimates that there are 8 million new cases annually.^1^, ^2^ Cough, sputum with or without hemoptysis, fever, and constitutional symptoms are the hallmark manifestations of an active tuberculosis infection. In patients with pulmonary TB, an increase in hemoglobin levels is regarded as an indicator of a positive response to treatment. Furthermore, Omar et al. found that a fall in platelet count, white blood cell (WBC) count, and erythrocyte sedimentation rate (ESR) were strong indications of clinical response.^3^ Despite the possibility of an increase in white blood cells (WBC), which results in lymphocyte predominance, in clinical practice, eosinophilia is a usual finding that is self‐limiting in moderate cases, but it is exceedingly infrequent in TB.^4^, ^5^

Many allergic, viral, and neoplastic conditions may produce peripheral blood eosinophilia, necessitating a variety of examinations and subsequent therapy. Common causes of eosinophilia in children include infections with helminthic parasites, allergic diseases, malignancies, and adverse drug reactions.^1^, ^6^ One of the primary goals of the early evaluation is to identify an underlying cause that needs specific therapy. Even though difficulties linked with eosinophilia are more prevalent in individuals with higher eosinophil counts (>1500 eosinophils/uL), the peripheral blood eosinophil count does not accurately assess the risk of organ damage in each patient. A patient with modest peripheral blood eosinophilia may also have significant eosinophil organ involvement. Normal eosinophil counts in the human blood range between 0 and 350/mm3. This quantity accounts for between 1 and 3% of the differential leukocyte count.^7^ Most reports of eosinophilia in tuberculosis describe local eosinophilia as opposed to peripheral eosinophilia.^8^ To the best of our knowledge, reported cases are very rare. As a consequence, we describe one case of TB with considerable peripheral eosinophilia and the treatment outcome.

## 2 CASE PRESENTATION

A 9‐year‐old male child was presented to the pediatrics department with his mother, reporting symptoms of fever and persistent cough of 1 month duration, associated with acute respiratory distress. The fever was sudden in onset, low grade, and associated with chills. There were no aggravating factors, and it was alleviated through the use of medication. The child was active during the period between febrile episodes. The cough was productive, not associated with aggravating factors, and not relieved by medications. A history of weight loss and loss of appetite were present. There were no similar complaints among his family members.

The patient had a past medical history of fever and myalgia, which was diagnosed as typhoid fever. He had been given all the scheduled immunizations, including BCG, without experiencing any significant adverse effects.

On examination, the patient was thin‐built, not active, and oriented to time, place, and person. The patient was tachypneic (38/min), afebrile, and hemodynamically stable. Cervical lymph nodes were palpable, nontender, firm, and 2–3 in number on both sides. A chest examination revealed bilateral crepitations in the mammary, inframammary, axillary, and infra‐axillary regions. Other systems revealed no abnormalities.

Initial blood investigations (Table 1) revealed a total leucocyte count (TLC) of 36,000 cells per cubic mm, a differential count of neutrophils of 11%, lymphocytes (13%), and eosinophils of 72%. A peripheral smear showed normocytic normochromic blood with eosinophilic leukocytosis. The absolute eosinophil count (AEC) was 25,920 cells per cubic mm. Liver function tests (LFT) and renal function tests (RFT) were normal. Hepatitis B surface antigen, anti‐hepatitis C antibody, and human immunodeficiency virus (HIV‐1 and HIV‐2) ELISA results were all negative. Mantoux's skin test was negative. He underwent additional tests, including a peripheral smear for microfilaria, the Widal test, the Dengue NS1 antigen test, toxoplasmosis serology, and sputum fungal staining, all of which were negative. Blood, urine, and stool cultures were also negative. The bone marrow aspiration performed to rule out eosinophilic leukemia showed a negative result.

**TABLE 1:** Routine blood investigations on admission.

Test	Result	Units	Normal range	Method
Hemoglobin	11.6	gm/dL	11.5–15.5	Colorimetric
Total count	**36,000**	cells/cu mm	5000–13,000	Impedance
Neutrophils	**11**	%	40–80	Light Microscopy
Lymphocytes	**13**	%	20–40	Light Microscopy
Eosinophils	**72**	%	01–06	Light Microscopy
Monocytes	04	%	02–10	Light Microscopy
Basophils	00	%	0–2	Light Microscopy
Packed cell volume (PCV)	35.7	Vol %	35–45	Calculation
Mean corpuscular volume (MCV)	87.5	Fl	77–95	Calculation
Mean corpuscular hemoglobin (MCH)	28.4	Pg	25–33	Calculation
Mean Corpuscular Hemoglobin Concentration (MCHC)	32.5	%	31.0–37.0	Calculation
Red blood cell count	4.08	millions/cu mm	4.0–5.2	Impedance
Platelet count	4.4	lakhs/cu mm	1.4–4.1	Impedance

*Note:* Bold indicates eosinophil infiltration through numbers and result.

**FIGURE 1:**
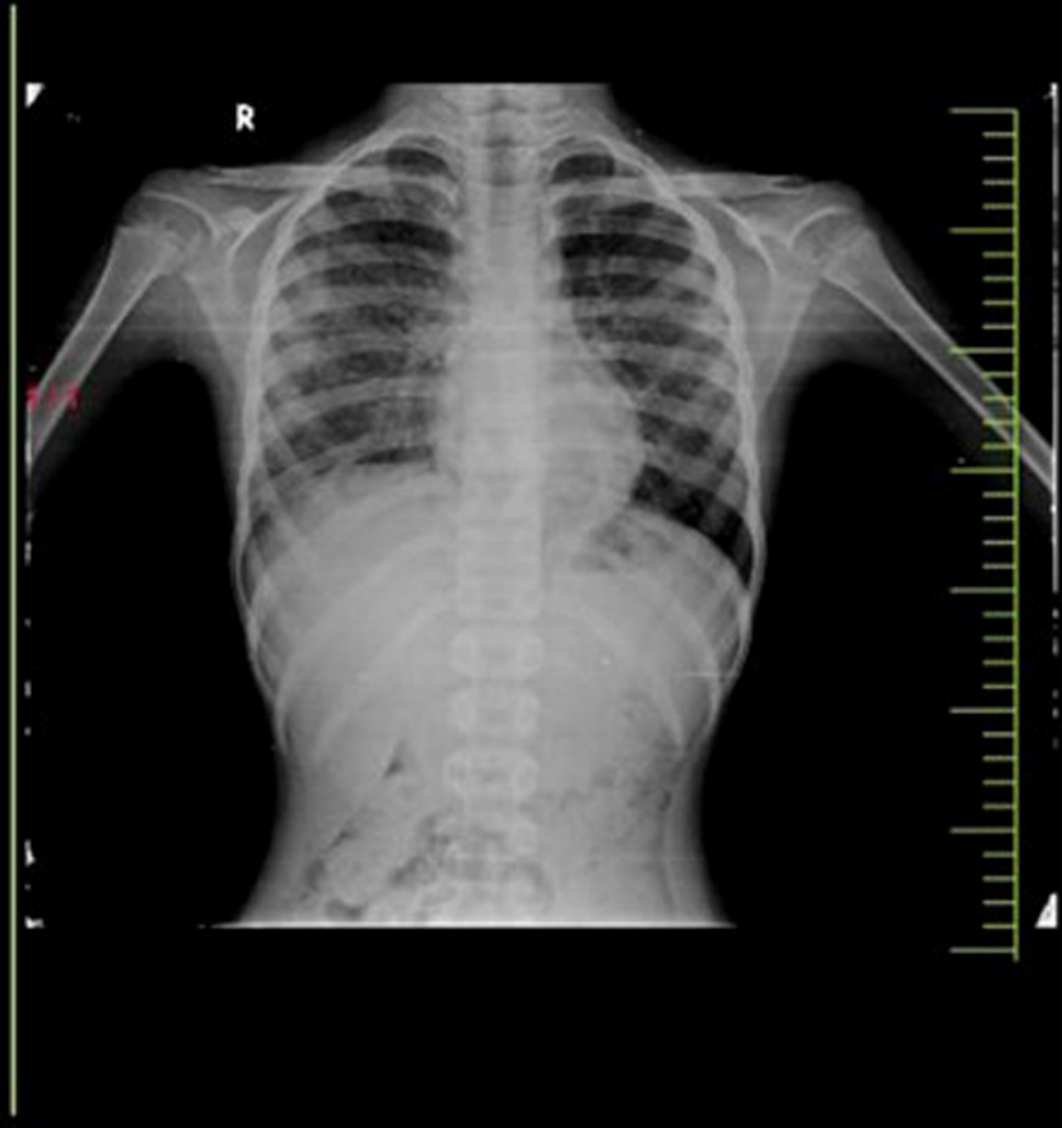
Chest x‐ray showing diffuse nodular calcifications in both lungs, involving all zones.

**FIGURE 2:**
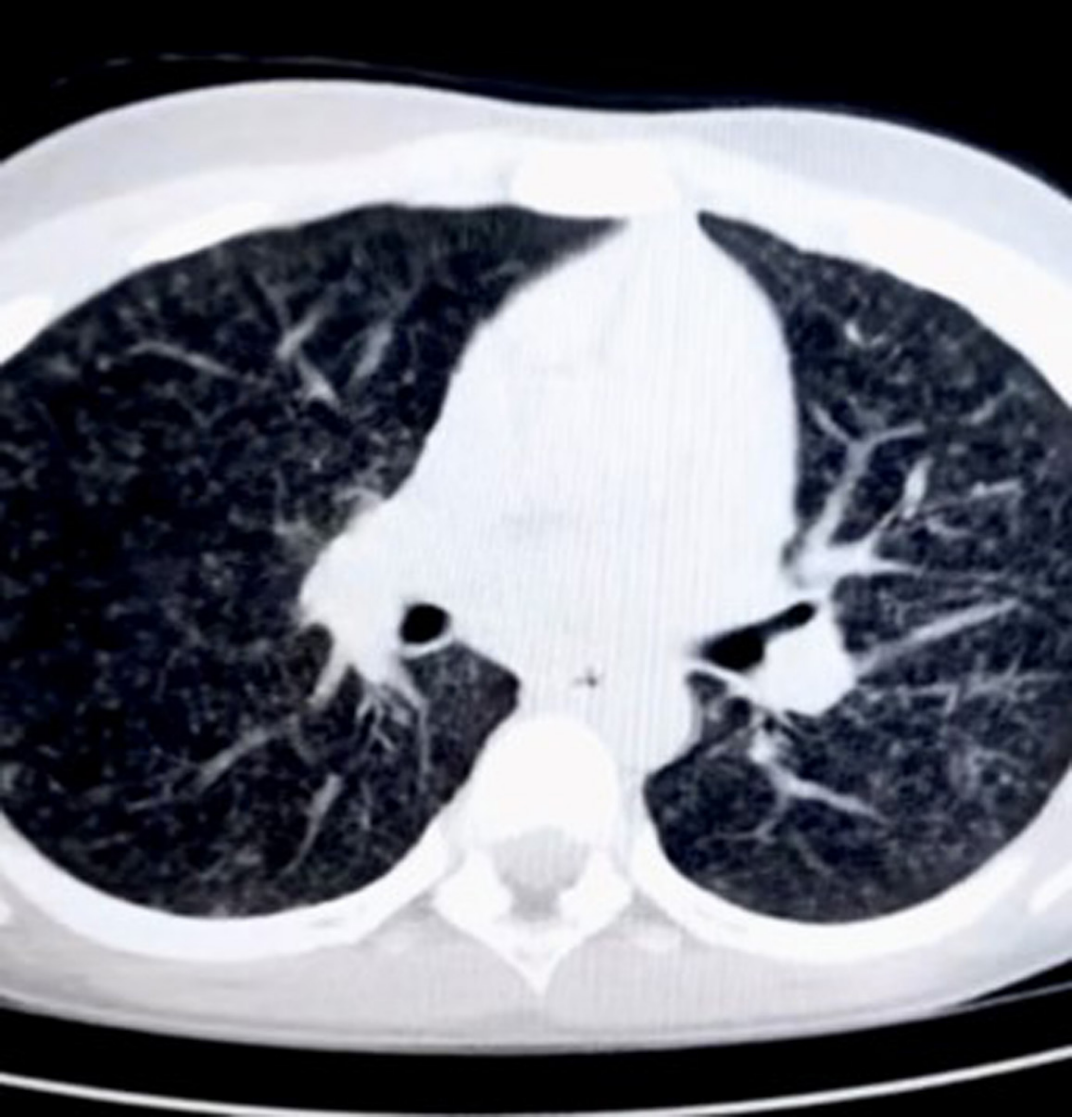
HRCT showing numerous tiny (2–3 mm) nodules in both lungs, with branching patterns in both the upper and lower lobes.

A chest x‐ray revealed diffuse nodular calcifications in bilateral lungs involving all zones (Figure 1). High‐resolution computed tomography (HRCT) chests show multiple tiny (2–3 mm) centrilobular and peri‐broncho‐vascular branching nodular densities noted scattered diffusely and equally in bilateral lungs, both in the upper and lower lobes (Figure 2). Ultrasound (US) of the chest showed mild left‐sided pleural effusion with lung consolidation and collapse. The US neck detected sub‐centimetrically enlarged cervical lymph nodes (7–8 mm). ZN stain and cartridge‐based nucleic acid amplification test (CBNAAT) tests on sputum samples produced normal results.

The child was initially started on amoxicillin and clavulanic acid (a combination antibiotic). Due to his raised eosinophil count, the child was given diethylcarbamazine. Other symptomatic and supportive treatment was given with intravenous fluids, pantoprazole, paracetamol, lactic acid bacillus (a probiotic), and nebulizations with 3% normal saline and levo salbutamol. Chest physiotherapy and spirometric exercises were advised. In view of radiologically diagnosed pulmonary tuberculosis, the patient's regimen shifted to antitubercular therapy (ATT) with a fixed‐dose combination of isoniazid, rifampicin, pyrazinamide, and ethambutol with pyridoxine. Complaints of coughing and difficulty breathing subsided at the time of discharge. At 1‐month follow‐up, his eosinophil counts returned to normal levels.

## 3 DISCUSSION

The most prevalent reasons for increased eosinophil count are parasitic infections, allergy/atopy, urticaria, eczema, allergic rhinitis, angioneurotic edema, reactive eosinophilia subsequent to T‐cell lymphoma, acute leukemia, B‐cell lymphoma, eosinophilic leukemia, allergic drug reactions, idiopathic hyper‐eosinophilic syndrome, and collagen vascular diseases such as rheumatoid arthritis, eosinophilic fasciitis, or allergic angiitis.^9^

Although TB is not a well‐known cause of eosinophilia in the medical literature, it was identified in our case. Based on current evidence, there have been few reported instances of this phenomenon in recent years. Flores et al. described a similar case of peripheral blood eosinophilia and TB, which included symptoms such as lethargy, weight loss, and lymphadenopathy and was confirmed by a lymph node biopsy that revealed a granulomatous lesion.^10^ Gill et al. reported a case of abdominal tuberculosis with peripheral eosinophilia. The patient was diagnosed with abdominal tuberculosis based on a histopathological examination of peritoneal tissue.^8^ Similarly, Garg et al. reported a case of peripheral blood eosinophilia and TB, which included symptoms such as cough, generalized weakness, and significant weight loss and was confirmed by endoscopic bronchial ultrasound‐guided fine‐needle aspiration of the mediastinal lymph node that revealed acid‐fast bacilli.^5^

Ray et al. proposed that after experiencing an early hypersensitive reaction to the Mycobacterium antigen, susceptible people can develop florid tropical pulmonary eosinophilia.^11^ IL‐5 has been shown to be the main cytokine driving the development of peripheral eosinophilia in people with pulmonary TB. The release of toxic eosinophil products is closely associated with tissue pathology. Examples of such by‐products are eosinophil cationic protein, major basic protein, and eosinophil‐derived neurotoxin. Potentially, the emission of reactive oxygen species may cause tissue injury.^12^ The normal range of eosinophils in blood is between 0.0 and 6.0%, and between 30 and 350 is the typical range for the AEC. AEC between 0.5 and 1.0 × 109/L (SI units) or 0.5 and 1.0 × 103 cells/microliter (conventional units) is considered to be mild blood eosinophilia, whereas AEC greater than or equal to 1.5 × 109/L is considered to be hyper‐eosinophilia.^13^ Our patient presented with an AEC of 70%, accounting for 25,920 cells, and a TLC of 36,000.

Hsu et al. reported that patients undergoing peritoneal dialysis had modest peripheral blood and peritoneal fluid eosinophilia. The eosinophilia persisted despite the cessation of dialysis, but it disappeared following the initiation of antituberculous treatment.^14^ Similarly, Haftu et al. reported a rare case of hepatic TB with significant peripheral eosinophilia.^15^ Our patient's imaging results aided in clinching the diagnosis. Further evidence supporting the link between TB and peripheral eosinophilia is the patient's positive clinical response to anti‐TB therapy with Ethambutol, Rifampicin, Isoniazid, and Pyrazinamide for the initial two months, followed by rifampicin and isoniazid for the next 4 months (2ERHZ/4RH). Patients with peripheral eosinophilia should have TB considered as a differential diagnosis, particularly after more prevalent reasons have been ruled out.

A few similar cases in the literature are described below (Table 2).

**TABLE 2:** List of similar cases published in literature.

Author	Case age/Sex	Sign/symptoms	Radiographic findings	Laboratory findings	Treatment plan	Outcome
Flores et al, 1983^1^	61/M	Skin lesions, mild splenomegaly, cervical, axillary, and inguinal lymphadenopathy, fever, weakness, fatigue, and weight loss.	Abdominal computed tomography (CT) revealed retroperitoneal lymphadenopathy.	‐White Blood cell (WBC), 8200/cu mm, 26% neutrophils, 12% lymphocytes, 62% eosinophils; ‐Biopsies of skin lesions, liver, and lymph nodes revealed caseating granulomas with Langhans' giant cells and eosinophils. ‐A Ziehl–Neelsen (ZN) stain found acid‐fast bacteria. **‐A needle‐aspirated bone marrow specimen showed eosinophil infiltration.**	Isoniazid, rifampin, ethambutol, streptomycin	The patient's health improved quickly.
Gill et al, 1940^8^	19/M	Sharp chest pain, dry cough, sweating, upper abdominal discomfort, fever, and tachycardia.	Right‐sided pleural effusion.	‐A chest fluid sample was cloudy and blood‐stained, and a differential count indicated 80% eosinophil polymorphonuclear leukocytes. ‐Blood eosinophils 14%.	Laparotomy	The patient died 11 weeks after admission as his health worsened.
Hsu et al, 2000^14^	66/F	Chronic renal failure, bilateral neck mass growth (1–3 cm) and malaise.	CT showed numerous lymphadenopathies and substantial central necrosis on both neck sides.	Peritoneal eosinophilia (54%–85%) and peripheral eosinophilia (7%–12%), pus‐like material with unidentified non‐fermentative Gram‐negative bacilli in the biopsy.	Isoniazid, rifampicin, ethambutol, and pyrazinamide.	About 2 weeks later, her peripheral and peritoneal fluid eosinophilia subsided.
Garg et al, 2017^5^	68/F	Cough, fatigue, weight loss, skinny, and pale.	A contrast‐enhanced CT of the chest and abdomen showed enlarged non‐necrotic mediastinal lymph nodes with chronic liver disease features.	‐Neutrophils 41%, lymphocytes 27%, and eosinophils 32%. ‐Needle‐aspirated bone marrow and biopsies showed increased eosinophilic. ‐Endoscopic bronchial ultrasound‐guided fine‐needle aspiration of mediastinal lymph nodes showed tubercular inflammation with bacilli‐positive acid‐fast stain.	Isoniazid, rifampicin, ethambutol, and pyrazinamide.	Eosinophil counts normalized within a week.
Haftu et al, 2020^15^	9/F	Right upper quadrant stomach ache, decreased appetite, vomiting, weight loss, lethargy, and low‐grade intermittent fever.	‐Abdominal ultrasound (US) shows hepatomegaly with aberrant echo pattern and cystic change. ‐Liver CT indicates several cystic and tiny daughter cysts as a connecting hypoechoic mass‐like lesion.	‐Leucocytosis with severe eosinophilia with 50%. ‐The biopsy showed partially encapsulated hepatocyte lobules with many epithelioid granulomas, large cells, necrosis, and micro abscesses and chronic inflammatory cells, mostly lymphocytes and eosinophils, confirming liver TB.	Isoniazid, rifampicin, ethambutol, and pyrazinamide.	After 6 months of antitubercular therapy (ATT), the patient was symptom‐free, gained 5 kg, and with normal lab findings.

*Note:* Bold indicates eosinophil infiltration through numbers and result.

## 4 CONCLUSION

The diagnostic process begins with a thorough history and physical examination. When collecting a patient's history, it is important to ask about travel history as well as any diseases or conditions related to collagen vascular tissue, changes in immunological function, medication use, or blockage of the airway. Even with infectious disorders, the ultimate diagnosis will always depend on how well the patient responds to therapy. In light of these findings, we believe that further studies are required to determine the pathophysiology of severe eosinophilia in TB and to identify this condition as a potential causal factor.

## AUTHOR CONTRIBUTIONS

**Abhiram Rao Damera:** Conceptualization; data curation; formal analysis; funding acquisition; investigation; writing – original draft; writing – review and editing. **Prakash Gupta:** Resources; software; supervision; validation; visualization; writing – original draft; writing – review and editing. **Shaheer Farooqi:** Resources; software; supervision; validation; visualization; writing – original draft; writing – review and editing. **Vivek Sanker:** Methodology; project administration; resources; software; supervision; validation; writing – original draft; writing – review and editing. **Arpita Mariam Mathews:** Resources; software; supervision; validation; visualization; writing – original draft; writing – review and editing. **Shreya Pampati:** Resources; software; supervision; validation; visualization; writing – original draft; writing – review and editing. **Manoj Reddy Allala:** Resources; software; supervision; validation; visualization; writing – original draft; writing – review and editing. **Tirth Dave:** Resources; software; supervision; visualization; writing – original draft; writing – review and editing.

## FUNDING INFORMATION

We did not receive any funding for this paper.

## CONFLICT OF INTEREST STATEMENT

The authors have no conflict of interest to declare.

## ETHICS STATEMENT

The ethical approval was not required for the case report as per the country's guidelines.

## CONSENT

Written informed consent was obtained from the patient to publish this report.

## Data Availability

The data that support the findings of this article are available from the corresponding author upon reasonable request.
